# One-step synthesis of PdCu@Ti_3_C_2_ with high catalytic activity in the Suzuki–Miyaura coupling reaction†

**DOI:** 10.1039/d2na00327a

**Published:** 2022-07-06

**Authors:** Dancheng Zhu, Kai Zheng, Jun Qiao, Hao Xu, Chao Chen, Pengfei Zhang, Chao Shen

**Affiliations:** Key Laboratory of Pollution Exposure and Health Intervention of Zhejiang Province, College of Biology and Environmental Engineering, Zhejiang Shuren University Hangzhou 310015 China shenchaozju@163.com; College of Material, Chemistry and Chemical Engineering, Key Laboratory of Organosilicon Chemistry and Material Technology, Ministry of Education, Hangzhou Normal University Hangzhou 311121 China

## Abstract

Owing to their enhanced catalytic stability and cyclability, two-dimensional (2D) material-supported Pd-based bimetallic alloys have promising applications for catalytic reactions. Furthermore, the alloying strategy can effectively reduce costs and improve catalytic performance. In this paper, we report a one-step reduction method to synthesize a novel heterogeneous catalyst, PdCu@Ti_3_C_2_, with good catalytic performance. The composition and structure of the as-prepared catalyst were characterized by inductively coupled plasma-mass spectrometry (ICP-MS), scanning transmission electron microscopy (STEM), energy-dispersive X-ray spectroscopy (EDX), and X-ray photoelectron spectroscopy (XPS). The catalyst particles, which were identified as a PdCu bimetallic alloy, exhibited good dispersion on the substrate. The performance of the catalyst in the Suzuki–Miyaura coupling reaction was studied, and the results showed that PdCu@Ti_3_C_2_ had excellent catalytic activity, similar to that of homogeneous Pd catalysts such as Pd(PPh_3_)_4_. Moreover, the prepared catalyst could be reused at least 10 times in the Suzuki–Miyaura coupling reaction with high yield.

## Abstract 2

Owing to their enhanced catalytic stability and cyclability, two-dimensional (2D) material-supported Pd-based bimetallic alloys have promising applications for catalytic reactions.

## Graphical abstract

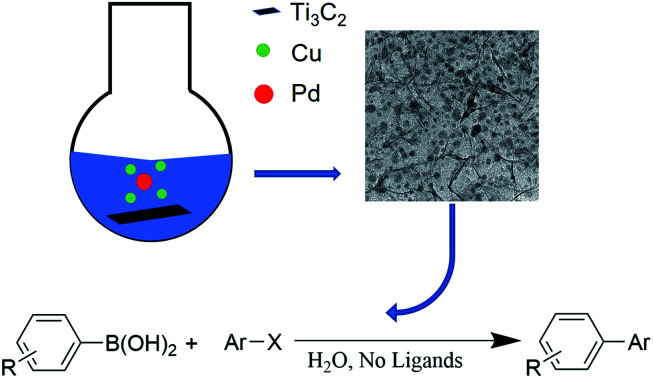

## Introduction

In recent years, two-dimensional (2D) materials have been widely used as carriers for the preparation of C–C coupling reactions because of their excellent properties.^1–5^ The use of 2D materials as carriers can effectively reduce the amount of Pd, a high-cost noble metal catalyst commonly used in C–C coupling reactions.^6–12^ However, the most widely used 2D substrate, graphene oxide (GO), cannot have both excellent electrical conductivity and hydrophilic properties simultaneously, and this limits the application of GO carriers. There are other problems with the use of GO as a substrate to prepare catalysts for C–C coupling reactions: (1) it requires surface functionalization^1,2^ or compounding with other materials^4^ to improve its charge transfer capability, making the catalyst preparation process more complicated; (2) the current catalysts have high catalytic activity only in organic solvents, which may have some environmental impact and loss of product;^1–5^ and (3) the high Pd content in the catalysts leads to high costs. Therefore, there is a need to develop a two-dimensional material with better performance as a catalyst carrier.

Ti_3_C_2_, a new 2D material, was first discovered in 2011.^13^ Compared to GO, Ti_3_C_2_ has the advantage of both excellent electrical conductivity and abundant surface functional groups,^14–21^ and it can be used as a substrate without complex functionalization processes. Ti_3_C_2_ has good hydrophilicity, which means that the prepared catalyst can use pure water as the reaction solvent, thereby reducing the impact on the environment and the loss of product. In addition, the catalytic activity can be effectively improved owing to the excellent properties of Ti_3_C_2_. As an advantage compared with previous reports,^22–29^ non-precious metals such as Cu can be added in the preparation of Pd nanoparticles (NPs) to prepare Pd-based nanoalloys or intermetallic materials and thereby reduce the cost of catalysts.

In this paper, we present a one-step synthetic method to grow Pd/Cu NPs on Ti_3_C_2_ nanosheets (PdCu@Ti_3_C_2_). This method has the advantages of simple operation and high utilization of the metal precursors. Through a series of characterization studies, these particles were identified as Pd/Cu bimetallic alloys (1 : 4 molar ratio) with good dispersion on the substrate. PdCu@Ti_3_C_2_ showed high catalytic activity in Suzuki–Miyaura coupling reactions in aqueous environments without ligands. We also demonstrated that these catalysts could be recycled and reused more than ten times without significant change in catalytic efficiency.

## Experimental section

### Chemicals and materials

MAX (Ti_3_AlC_2_) powder was purchased from Rhawn Chemical Technology Co. Ltd, hydrofluoric acid (HF, 40%) from Sinopharm Chemical Reagent Co., polyvinylpyrrolidone (PVP) and palladium diacetate (Pd(Ac)_2_) from Shanghai Aladdin Biochemical Technology Co., Ltd, ethylene glycol (EG, 99%) from Lingfeng Chemical Reagent Co., and cupric acetate (Cu(Ac)_2_) from Chembee Chemical Co. All chemicals and materials were used without further purification.

### Preparation of MXenes (Ti_3_C_2_)

Ti_3_C_2_ MXenes were prepared *via* chemical etching of the as-received Ti_3_AlC_2_ powders, as listed below. First, 2 g of Ti_3_AlC_2_ powder was added to 20 mL of HF solution and stirred at room temperature for 24 h, and the solid was then separated by centrifugation (3500 rpm) and washed with deionized (DI) water until the pH of the supernatant solution was >6. The collected solid was finally dried in a vacuum for another 24 h, yielding the desired product Ti_3_C_2_ as a black powder.

### Preparation of PdCu@Ti_3_C_2_

The as-synthesized Ti_3_C_2_ powder (200 mg) was added to 50 mL of EG, and the mixture was ultrasonicated for 6 h. Pd(Ac)_2_ (22 mg), Cu(Ac)_2_ (72 mg), and PVP (300 mg) were added, and the mixture was stirred at 170 °C in an oil bath for 2 h. The mixture was then centrifuged at 4500 rpm, and the solid residue was washed several times with ethanol and DI water and finally freeze-dried to give the product PdCu@Ti_3_C_2_ as a powder. For comparison purposes, Pd^1^@Ti_3_C_2_, Pd^2^@Ti_3_C_2_ and Cu@Ti_3_C_2_ were also prepared following the same protocol, the corresponding precursor amounts being 22 mg of Pd(Ac)_2_, 110 mg of Pd(Ac)_2_, and 90 mg of Cu(Ac)_2_ respectively, with the other quantities remaining unchanged.

### Morphological and structural characterization

Inductively coupled plasma-mass spectrometry (ICP-MS) was performed using an Agilent 7700. Scanning electron microscopy (SEM) was performed using a Hitachi SU-70. Transmission electron microscopy (TEM) images were obtained using an FEI Tecnai G2 F20 microscope operated at 200 kV. Scanning transmission electron microscopy (STEM) was performed using an FEI Chemi-STEM Titan G2 80-200 equipped with a probe-side spherical aberration corrector and operated at an acceleration voltage of 200 kV. Energy-dispersive X-ray spectroscopy (EDS) was performed using a Bruker super-X detection system. X-ray diffraction (XRD) spectroscopy was performed using a Rigaku SmartLab SE diffractometer. X-ray photoelectron spectroscopy (XPS) was performed using a Thermo Scientific K-Alpha instrument with monochromatic Al_Kα_ radiation.

### General procedure for the Suzuki coupling reactions

4-Iodoanisole (0.5 mmol), phenylboronic acid (0.65 mmol), K_2_CO_3_ (1 mmol), PdCu@Ti_3_C_2_ (10 mg), and H_2_O (5 mL) were placed in a reaction flask and stirred at 80 °C for 1–3 h. After the reaction, the mixture was extracted with ethyl acetate. The organic layer was dried with magnesium sulfate, filtered, and concentrated *in vacuo*. The residue was purified by silica gel chromatography using petroleum ether to obtain the desired product.

### General procedure for catalyst recovery

4-Iodoanisole (2.5 mmol), phenylboronic acid (3.25 mmol), K_2_CO_3_ (5 mmol), PdCu@Ti_3_C_2_ (50 mg), and H_2_O (25 mL) were placed in a reaction flask and stirred at 80 °C for 1 h. After the reaction, ethyl acetate, ethanol, and deionized water were added successively for centrifugal cleaning. The residual catalysts were then freeze-dried.

## Results and discussion

**Fig. 1:**
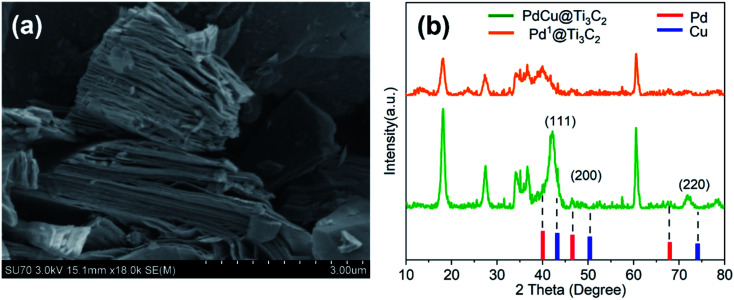
(a) SEM image of Ti_3_C_2_; (b) XRD spectra of PdCu@Ti_3_C_2_, Pd^1^@Ti_3_C_2_.

We introduced a one-step reduction method to synthesize PdCu@Ti_3_C_2_ NPs. The samples of Ti_3_AlC_2_ were etched with HF for 24 h to produce Ti_3_C_2_ powders. Significant delamination was observed (Fig. 1a), indicating that the Al atoms in Ti_3_AlC_2_ had been etched out. Ultrasonic dispersion of the powder in EG solution led to the formation of a monolayer or a few layers of two-dimensional Ti_3_C_2_ as the catalyst carrier. Next, the metal precursor (Pd(Ac)_2_ or Cu(Ac)_2_) and the surfactant (PVP) were added to the mixture, and the precursor was reduced by EG at 170 °C as described in a previous study.^30^ The loading of PdCu@Ti_3_C_2_ was characterized by ICP-MS (Table S1†). The mass fractions of Cu and Pd in the PdCu@Ti_3_C_2_ sample were 9.16% and 3.59%, respectively, compared with the theoretical mass fractions of 10.8% and 4.5%, respectively. The small differences and the small amount of residual PVP during the synthesis suggest that the preparation method had high utilization of the metal precursors.

The structures of the PdCu@Ti_3_C_2_ and Pd^1^@Ti_3_C_2_ powders were characterized by XRD (Fig. 1b). Comparison with the diffraction pattern in a previous paper^13^ shows that the main peaks of the two materials match those of Ti_3_C_2_ after HF etching. Further analysis of PdCu@Ti_3_C_2_ revealed that the intensity of the peak at 42° was significantly higher than that of Pd^1^@Ti_3_C_2_, and two additional peaks were observed, at 47°and 73°. Comparison with the PDF cards of Pd (PDF#88-2335) and Cu (PDF#85-1326) showed that these diffraction peaks were located between the (111), (200), and (220) peaks of Pd and Cu, respectively, indicating that the prepared NPs were bimetallic alloy materials.

**Fig. 2:**
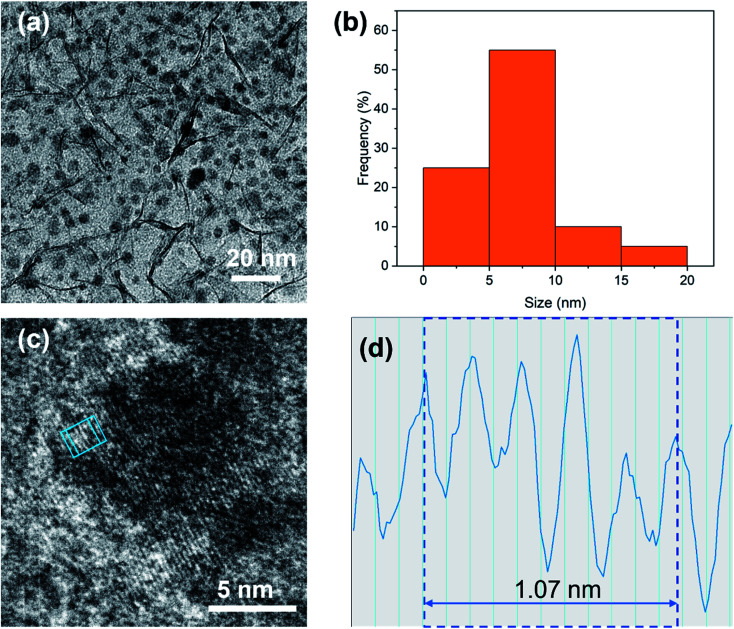
(a) Low magnification TEM image of PdCu@Ti_3_C_2_. (b) The size distribution of ultrafine PdCu NPs. (c) HRTEM image of PdCu NPs. (d) The profile of the blue box in the HRTEM image.

TEM was used to further characterize the microstructure of PdCu@Ti_3_C_2_ (Fig. 2). Some NPs were distributed on the surface of the Ti_3_C_2_ nanosheets (Fig. 2a). Statistically, most of these particles were approximately 5–10 nm in size and exhibited good dispersion. Only a small percentage of the particles agglomerated (Fig. 2b). Individual NPs were characterized using high-resolution (HR)TEM to identify their structures (Fig. 2c). These NPs have ordered lattice strips with a lattice spacing of 0.214 nm (Fig. 2d), which is slightly larger than the spacing of the (111) crystal planes of face-centered cubic Cu (0.206 nm). A small number of Pd atoms entered the Cu lattice with a spacing of 0.225 nm between the (111) planes, leading to an increase in the crystalline spacing of the NPs.

**Fig. 3:**
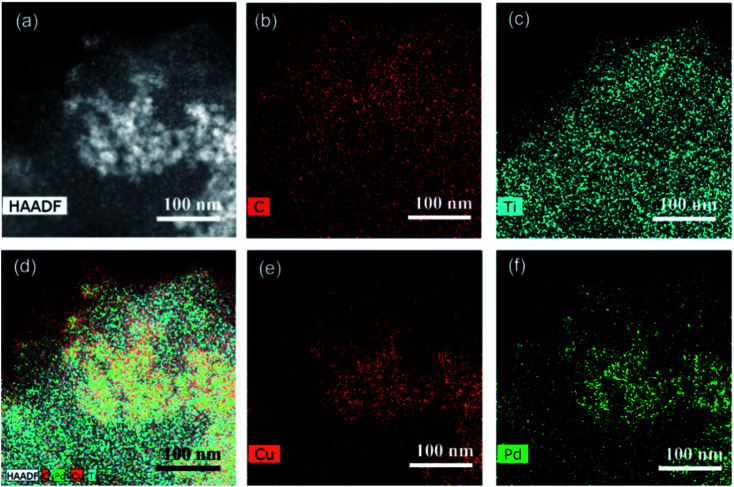
(a) STEM image of PdCu nanoparticles. (b–f) EDS mapping of PdCu@Ti_3_C_2_. (b) C, (c) Ti, (e) Cu, and (f) Pd.

Subsequently, the elemental composition and distribution of PdCu@Ti_3_C_2_ were analyzed using STEM. As shown in the annular dark-field (ADF) STEM images and EDS mapping images (Fig. 3), Ti and C were evenly distributed in the sample area, indicating that the substrate material was Ti_3_C_2_ (Fig. 3b and c). Cu and Pd were concentrated in the brighter area in the middle of the ADF-STEM image, with similar distributions (Fig. 3e and f). This further indicates the successful preparation of a bimetallic alloy structural material. The ICP characterization results of Pd^1^@Ti_3_C_2_, Pd^2^@Ti_3_C_2_, and Cu@Ti_3_C_2_ verified this conclusion (Table S1†). The loading of Pd on both Pd^1^@Ti_3_C_2_ and Pd^2^@Ti_3_C_2_ was found to be only 1% by ICP characterization, which is much smaller than the amounts of the metal precursor. The mass fraction of Cu on Cu@Ti_3_C_2_ reached 11.43%, which is similar to the amount of the metal precursor. The above results show that Pd is difficult to load on Ti_3_C_2_ nanosheets. This is mainly because oxygen-containing groups are critical for the loading ability of Pd. The main functional group on the surface of Ti_3_C_2_ was F^−^, and the number of oxygen-containing functional groups was low, which made it difficult for the Pd monomers to be directly loaded on Ti_3_C_2_. The formation of a PdCu alloy can effectively increase the loading of Pd.

**Fig. 4:**
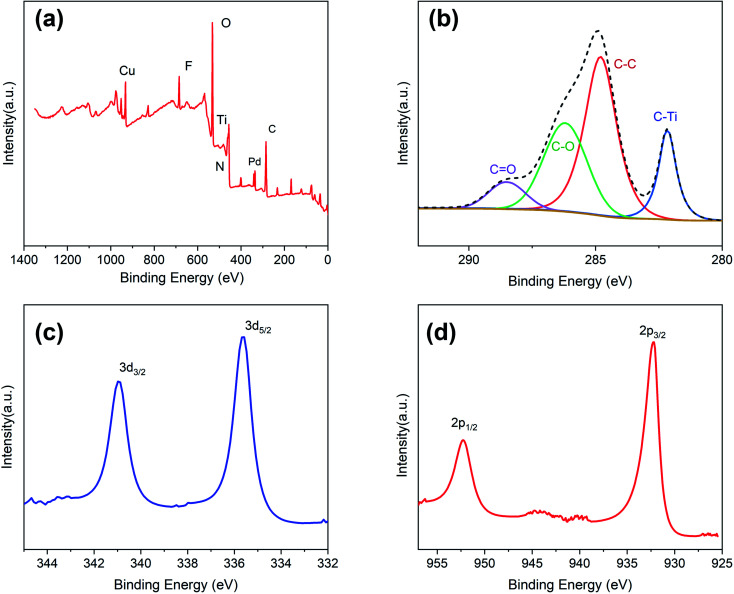
XPS spectra of PdCu@Ti_3_C_2_ nanosheets (a) wide, (b) C1s, (c) Pd3d, and (d) Cu2p.

The chemical and electronic states of C, Pd, and Cu were analyzed by XPS (Fig. 4). During the preparation of Ti_3_C_2_ nanosheets, a large number of functional groups are generated on the surface, such as OH^−^, O^2−^, and F^−^.^13^ Their presence was verified by XPS (Fig. 4a and b), and these groups play an important role in the growth of the PdCu alloy NPs. Fig. 4c shows the XPS spectrum of the Pd 3d core layer with the Pd 3d_5/2_ and Pd 3d_3/2_ components at 335.6 and 340.9 eV, respectively. Both peaks originating from Pd are assigned to the Pd (0) state. Comparison with the data in the manual shows a shift of approximately 0.3 eV in the two Pd peaks. This chemical shift is due to a strong interaction between Pd and Cu. The XPS spectrum of the 2p orbitals of Cu also supports this speculation (Fig. 4d). The 2p_1/2_ and 2p_3/2_ orbitals of Cu are located at 952.23 and 932.2 eV respectively, with a shift of 0.4 eV towards lower binding energy. This phenomenon indicates an increase in the electron cloud density around the PdCu NPs, which improves the catalytic performance.

After characterizing the structure and chemical properties of PdCu@Ti_3_C_2_, we evaluated the catalytic efficiency of the as-prepared catalysts in the Suzuki coupling reaction (Table 1). 4-Iodoanisole and phenylboronic acid were used as model substrates, and water was used as the solvent. Several different catalysts, including Ti_3_C_2_, PdCu, PdCu@Ti_3_C_2_, Pd^1^@Ti_3_C_2_, Pd^2^@Ti_3_C_2_, and Cu@Ti_3_C_2_, were investigated in the reaction. Pure Ti_3_C_2_ had negligible catalytic activity (entry 1), and the catalyst loaded with only Cu (Cu@Ti_3_C_2_) had low catalytic activity (entry 3). Catalysts loaded with Pd showed significantly higher efficiencies, with reaction yields higher than 80%. The PdCu@Ti_3_C_2_ catalyst resulted in the highest reaction yield of 95%, which was higher than those of Pd^1^@Ti_3_C_2_ and Pd^2^@Ti_3_C_2_ (entries 4 and 5). The use of Ti_3_C_2_ as the carrier also improved the catalyst efficiency, as the use of PdCu NPs as catalysts decreased the yield to 87% (entry 2). We also varied other reaction parameters, such as time, temperature, and base (entries 6 and 8–13). When the reaction time was increased from 0.5 to 1 h, a higher reaction yield was obtained. Further increasing the reaction time to 3 h resulted in a negligible difference in the reaction yield (entries 6, 8, and 9). Therefore, the optimal reaction time was 1 h. Similarly, we found that the optimal reaction temperature was 80 °C, as the reaction yield increased with increasing temperature up to but not above 80 °C (entries 6, 10, 11). We also tried different bases, and the best yield was obtained using K_2_CO_3_ (entries 6, 12, and 13). The catalytic activity of PdCu@Ti_3_C_2_ was compared with those of heterogeneous catalysts reported in the literature (Table 2). PdCu@Ti_3_C_2_ exhibits excellent catalytic activity at relatively mild temperatures in pure water.

**Table 1:** Optimization of reaction conditions^a^

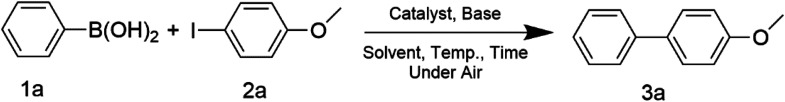					
Entry	Catalyst (mg)	Base	Temperature (°C)	Time (h)	Yield^b^ (%)
1	Ti_3_C_2_	K_2_CO_3_	80	1	NR
2	PdCu	K_2_CO_3_	80	1	87
3	Cu/MXene	K_2_CO_3_	80	1	33
4	Pd^1^/MXene	K_2_CO_3_	80	1	92
5	Pd^2^/MXene	K_2_CO_3_	80	1	92
6	PdCu/MXene	K_2_CO_3_	80	1	95
7	PdCu	K_2_CO_3_	80	1	87^c^
8	PdCu/MXene	K_2_CO_3_	80	3	95
9	PdCu/MXene	K_2_CO_3_	80	0.5	92
10	PdCu/MXene	K_2_CO_3_	70	1	74
11	PdCu/MXene	K_2_CO_3_	90	1	96
12	PdCu/MXene	KOH	80	1	87
13	PdCu/MXene	NaOH	80	1	84

a The reaction conditions: phenylboronic acid 1a (0.65 mmol), 4-iodoanisole 2a (0.5 mmol), 10 mg catalyst, and base (1 mmol) in 5 mL H_2_O.

b Isolated yield.

c Catalyst mass is 5 mg.

**Table 2:** Comparison of PdCu@Ti_3_C_2_ with some recently reported Pd catalysts for the Suzuki coupling reaction

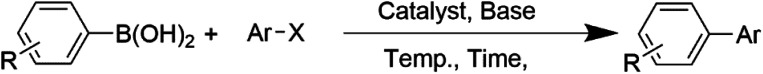							
Entry	R	Ar	X	Catalyst	Reaction condition	Yield (%)	Ref.
1	H	Ph	I	PdCu@Ti_3_C_2_	H_2_O/K_2_CO_3_/80 °C/1 h	97	This work
2	H	Ph	I	Pd@APGO	H_2_O : EtOH (1 : 1)/K_2_CO_3_/80 °C/6 h	96	1
3	H	Ph	I	Pd NCs	H_2_O : EtOH (1 : 1)/K_2_CO_3_/R.T./0.5 h	95	12
4	H	Ph	I	GO-TETA-Pd	H_2_O/Na_2_CO_3_/90 °C/10 min	85	3

**Fig. 5:**
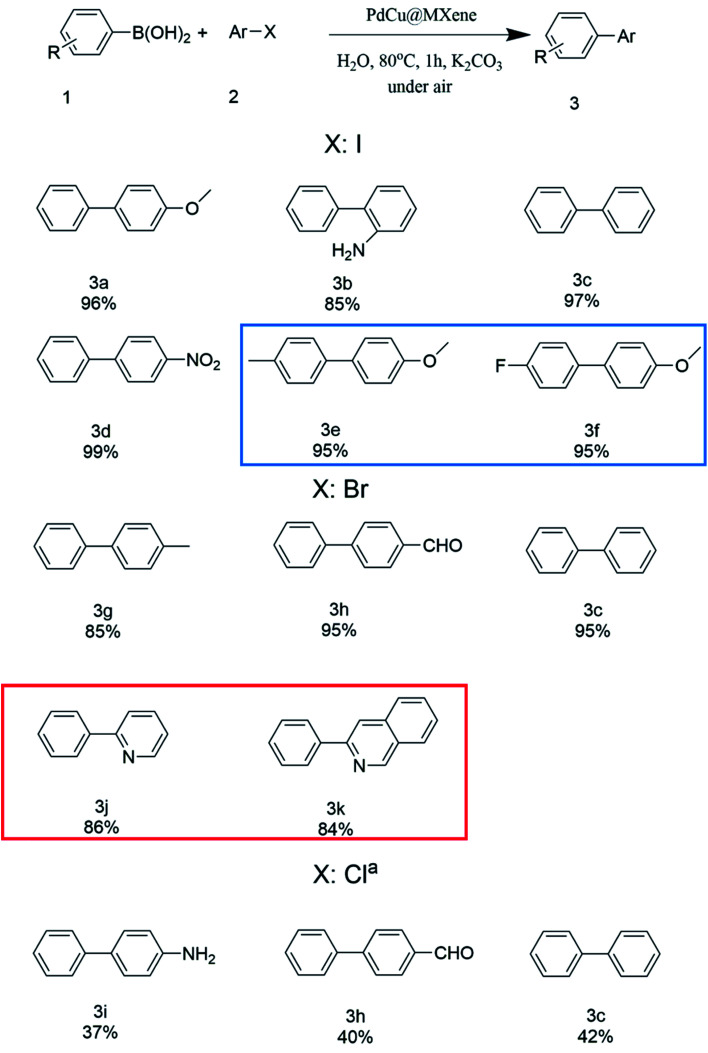
Suzuki coupling between phenylboronic acid and aryl halides. The reaction conditions: boronic acid 1 (0.65 mmol), aryl halide 2 (0.5 mmol), 10 mg PdCu@Ti_3_C_2_, and K_2_CO_3_ (1 mmol) in 5 mL water were reacted for 1 h in air. The blue box shows the products where different aryl boronic acids have been used as substrates. The red box shows the products using heteroaryl bromides as substrates. ^a^ 5 ml of a 1 : 1 mixture of water and toluene and 8 h.

To further explore the substrate scope using PdCu@Ti_3_C_2_ as the catalyst, various aryl halides were investigated under optimized reaction conditions (Fig. 5). Phenylboronic acid (1) was used as the substrate to test the effect of different aryl iodides. The yields of reactions between phenylboronic acids and iodobenzenes with both electron-withdrawing and electron-donating groups were 84–99%. The yields were higher when substrates with electron donating groups were used. Next, the impact of different halides was investigated. Under optimal reaction conditions, the coupling reaction of aryl bromides resulted in yields similar to those of aryl iodides. However, the reaction yields were significantly reduced when aryl chlorides were used as the substrates, even when the reaction time was extended to 8 h and the solvent was changed to a 1 : 1 mixture of water and toluene. The efficiency of the coupling reactions using different aryl boronic acids was investigated, and all gave high yields (3e, 3f). Finally, the reaction conditions were optimized for the Suzuki coupling of heteroaryl bromides with benzene boronic acids, and similar yields were obtained (3j, 3k).

**Scheme 1:**
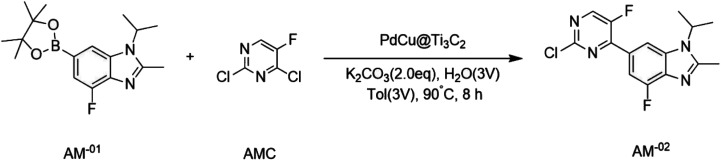
The application in the key intermediates for the synthesis of abemaciclib.

The PdCu@Ti_3_C_2_ catalyst was used to synthesize key intermediates for abemaciclib (Scheme 1),^31,32^ an FDA-approved drug used for the treatment of advanced or metastatic breast cancers. 2,4-Dichloro-5-fluoropyrimidine was used as a substrate for the synthesis of the intermediate AM^-02^. This transformation was completed with excellent results in the presence of the PdCu@Ti_3_C_2_ catalyst and K_2_CO_3_ at 90 °C for 6 h in a 1 : 1 mixture of water and toluene. Under these reaction conditions, the yield was 90%, which is higher than that previously reported.^31^

**Fig. 6:**
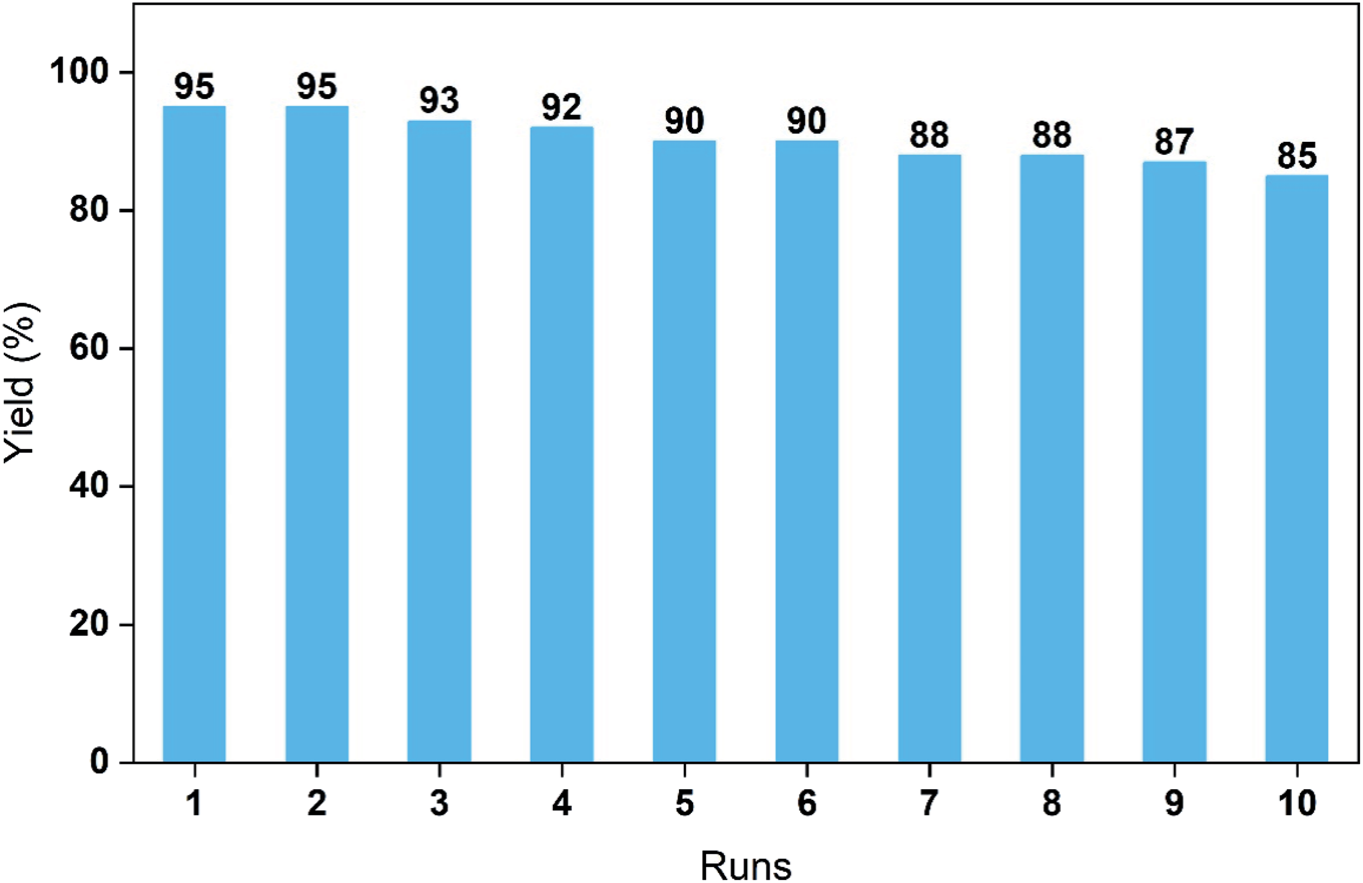
Recycling and reuse of PdCu@Ti_3_C_2_ in the Suzuki coupling.

Finally, the cycling performance of the PdCu@Ti_3_C_2_ catalyst was investigated. The catalytic performance was examined, based on the yields of the reaction between 4-iodoanisole and phenylboronic acid under the optimized conditions. The yield of the reaction decreased slightly after each run and remained at 85% after 10 cycles (Fig. 6). To further illustrate the performance and stability, we studied the structures of the recovered catalysts using TEM, STEM, and energy-dispersive X-ray spectroscopy (EDX) (Fig. S1 and S2†). As can be seen in Fig. S2a and b,† the morphology and structure of the catalyst after the first cycle were similar to those of the original catalyst. By the 10th cycle, the metal loading on the Ti_3_C_2_ surface was only slightly reduced; the metal structure had not changed, and there was only a small decrease in catalytic activity (Fig. S1c and d†). The ADF-STEM images and corresponding EDS mapping images of the catalysts after ten cycles indicated that the elements Pd and Cu were still evenly distributed. These results demonstrate the high stability of the PdCu@Ti_3_C_2_ NPs.

## Conclusion

In summary, we have successfully synthesized a new catalyst using a simple, one-step method. The structure of PdCu@Ti_3_C_2_ was studied, and the material was characterized. PdCu@Ti_3_C_2_ is an efficient heterogeneous catalyst for Suzuki coupling, and the optimal reaction conditions, including the base, temperature, and time, were explored. In addition, the catalysts could be recovered and reused at least ten times. The Suzuki reaction using this catalyst has the advantages of a green solvent, short reaction time, high yield, and no ligand requirement. Finally, this study expands the application of 2D Ti_3_C_2_ as a catalyst carrier and promotes the development of green chemistry.

## Conflicts of interest

There are no conflicts to declare.

## Supplementary Material

NA-004-D2NA00327A-s001
