# Supplementary material for: One-step synthesis of PdCu@Ti3C2 with high catalytic activity in the Suzuki–Miyaura coupling reaction
Source: Nanoscale Adv. 2022 Jul 6;4(16):3362–9. doi: 10.1039/d2na00327a (PMC9417861; doi:10.1039/d2na00327a)
Supplement: NA-004-D2NA00327A-s001 [file NA-004-D2NA00327A-s001.pdf]

Electronic Supplementary Material (ESI) for Nanoscale Advances.
This journal is © The Royal Society of Chemistry 2022

Supporting information for

Preparation and Characterization of PdCu@Ti_3C_2 Catalyst for Suzuki-Miyaura Coupling Reaction

Table S1. The content of Pd and Cu elements in the PdCu@Ti_3C_2, Pd^1@Ti_3C_2, Pd^2@Ti_3C_2

Sample	Element	Mass fraction（%)
PdCu@Ti_3C_2	Cu	9.16%
	Pd	3.59%
Pd^1@Ti_3C_2	Pd	1.40%
Pd^2@Ti_3C_2	Pd	1.02%
Cu@Ti_3C_2	Cu	11.43%

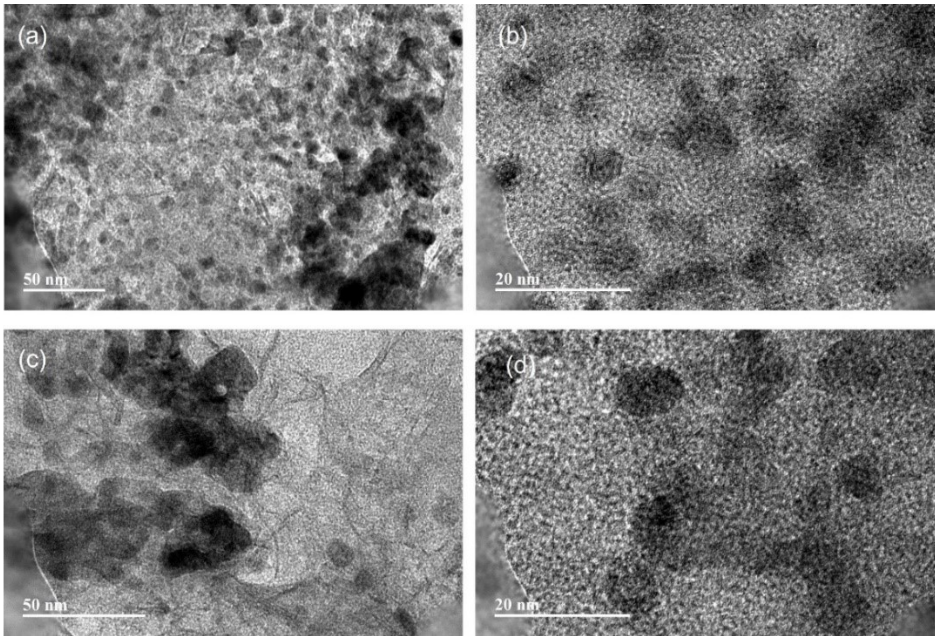

Figure S1. (a) Low magnification TEM image of PdCu@Ti_3C_2 after first cycle (b) HRTEM image of PdCu nanoparticles after first cycle. (c) Low magnification TEM image of PdCu@Ti_3C_2 after tenth cycle (d) HRTEM image of PdCu nanoparticles

after fifth cycle.

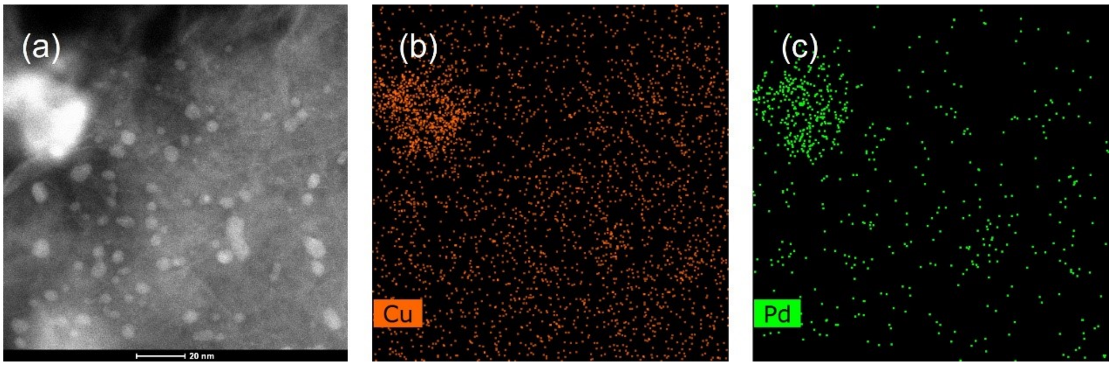

Figure S2. (a)STEM image of PdCu@Ti_3C_2 after tenth cycle. (b-f) EDS mapping of PdCu@Ti_3C_2 after cycle.

^{1}H and ^{13}C NMR spectra of the product

4-Methoxybiphenyl 3a:

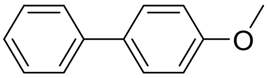

^{1}H NMR (500 MHz, $CDCl_3$) δ 7.54 (d, *J* = 7.5 Hz, 2H), 7.52 (d, *J* = 8.8 Hz, 2H), 7.40 (t, *J* = 7.7 Hz, 2H), 7.29 (t, *J* = 7.4 Hz, 1H), 6.97 (d, *J* = 8.7 Hz, 2H), 3.83 (s, 3H). **2-Aminebiphenyl 3b:**

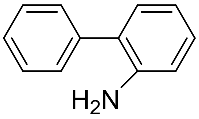

^{1}H NMR (600 MHz, $CDCl_3$) δ 7.43 (d, *J* = 8.2 Hz, 4H), 7.33 (t, *J* = 6.8 Hz, 1H), 7.15 – 7.11 (m, 2H), 6.82 (t, *J* = 7.4 Hz, 1H), 6.75 (d, *J* = 8.0 Hz, 1H), 3.63 (s, 2H).

Biphenyl 3c:

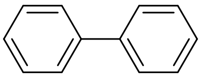

^{1}H NMR (500 MHz, $CDCl_3$) δ 7.56 (d, *J* = 7.1 Hz, 4H), 7.40 (t, *J* = 7.7 Hz, 4H), 7.31 (t, *J* = 7.4 Hz, 2H).

4-Nitrobiphenyl 3d:

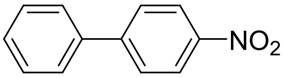

^{1}H NMR (500 MHz, $CDCl_3$) δ 8.30 (d, *J* = 8.9 Hz, 2H), 7.74 (d, *J* = 8.9 Hz, 2H), 7.62 (d, *J* = 7.1 Hz, 2H), 7.50 (t, *J* = 7.4 Hz, 2H), 7.44 (t, *J* = 7.3 Hz, 1H).

1-Methoxy-4-(4-methylphenyl)benzene 3e:

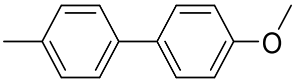

^{1}H NMR (500 MHz, $CDCl_3$) δ 7.52 (d, *J* = 8.8 Hz, 2H), 7.46 (d, *J* = 8.1 Hz, 2H), 7.24 (d, *J* = 7.9 Hz, 2H), 6.97 (d, *J* = 8.8 Hz, 2H), 3.85 (s, 3H), 2.39 (s, 3H).

1-Fluoro-4-(4-methylphenyl)benzene 3f:

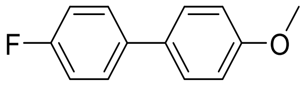

^{1}H NMR (500 MHz, $CDCl_3$) δ 7.52 – 7.46 (m, 4H), 7.14 – 7.08 (m, 2H), 7.00 – 6.96 (m, 2H), 3.86 (s, 3H).

4-Methylbiphenyl 3g:

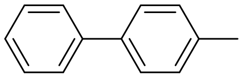

^{1}H NMR (500 MHz, $CDCl_3$) δ 7.57 (d, *J* = 8.5 Hz, 2H), 7.48 (d, *J* = 8.1 Hz, 2H), 7.41 (d, *J* = 7.9 Hz, 2H), 7.32 (d, *J* = 4.8 Hz, 1H), 7.22 (d, *J* = 7.9 Hz, 2H), 2.37 (s, 3H).

4-Phenylbenzaldehyde 3h:

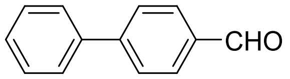

^{1}H NMR (600 MHz, $CDCl_3$) δ 10.06 (s, 1H), 7.95 (d, *J* = 8.0 Hz, 2H), 7.75 (d, *J* = 7.9 Hz, 2H), 7.64 (d, *J* = 7.4 Hz, 2H), 7.48 (t, *J* = 7.5 Hz, 2H), 7.42 (t, *J* = 7.3 Hz, 1H).

Biphenyl-4-amine 3i:

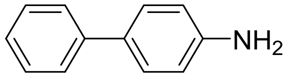

^{1}H NMR (600 MHz, $CDCl_3$) δ 7.53 (d, *J* = 7.1 Hz, 2H), 7.42 – 7.37 (m, 4H), 7.26 (d, *J* = 6.9 Hz, 1H), 6.73 (d, *J* = 7.6 Hz, 2H), 3.69 (s, 2H).

2-Phenylpyridine 3j:

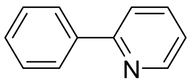

^{1}H NMR (600 MHz, $CDCl_3$) δ 8.69 (d, *J* = 4.6 Hz, 1H), 7.99 (d, *J* = 7.7 Hz, 2H), 7.72 (d, *J* = 6.9 Hz, 2H), 7.47 (t, *J* = 7.5 Hz, 2H), 7.41 (t, *J* = 7.3 Hz, 1H), 7.21 (t, *J* = 5.6 Hz, 1H).

3-Phenylquinoline 3k:

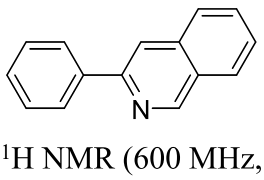

^{1}H NMR (600 MHz, $CDCl_3$) δ 9.17 (s, 1H), 8.25 (s, 1H), 8.14 (d, *J* = 8.4 Hz, 1H), 7.83 (d, *J* = 8.1 Hz, 1H), 7.70 – 7.66 (m, 3H), 7.54 (t, *J* = 7.5 Hz, 1H), 7.49 (t, *J* = 7.5 Hz,

2H), 7.41 (t, J = 7.4 Hz, 1H).

Key Intermediates for the Synthesis of Abemaciclib

^{1}H NMR (500 MHz, DMSO-d_6) δ 8.97 (d, J = 3.4 Hz, 1H), 8.16 (s, 1H), 7.63 (d, J = 11.8 Hz, 1H), 4.89 – 4.84 (m, 1H), 2.65 (s, 3H), 1.60 (d, J = 6.9 Hz, 6H).

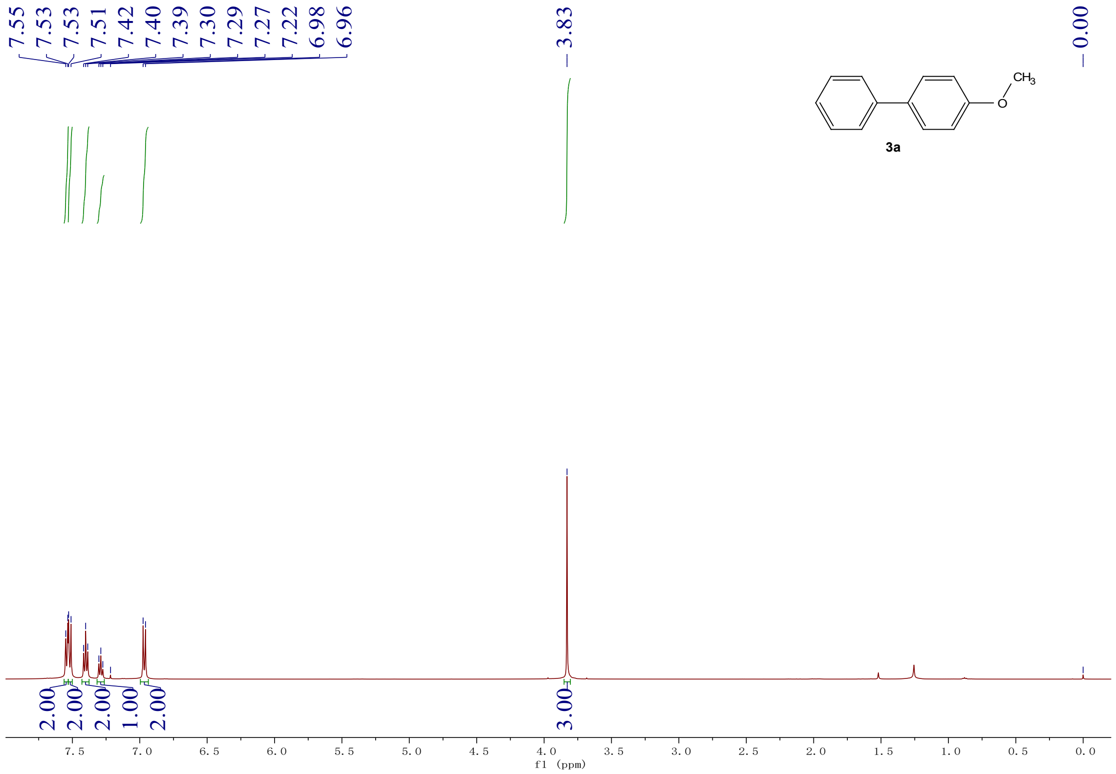

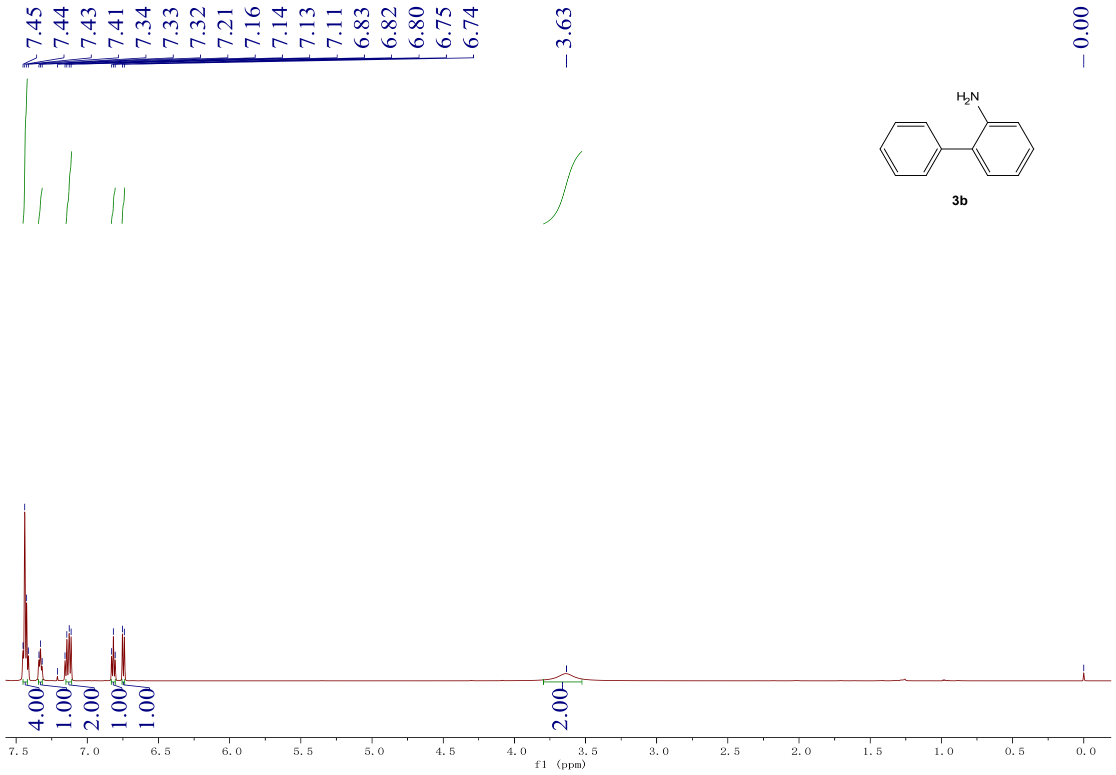

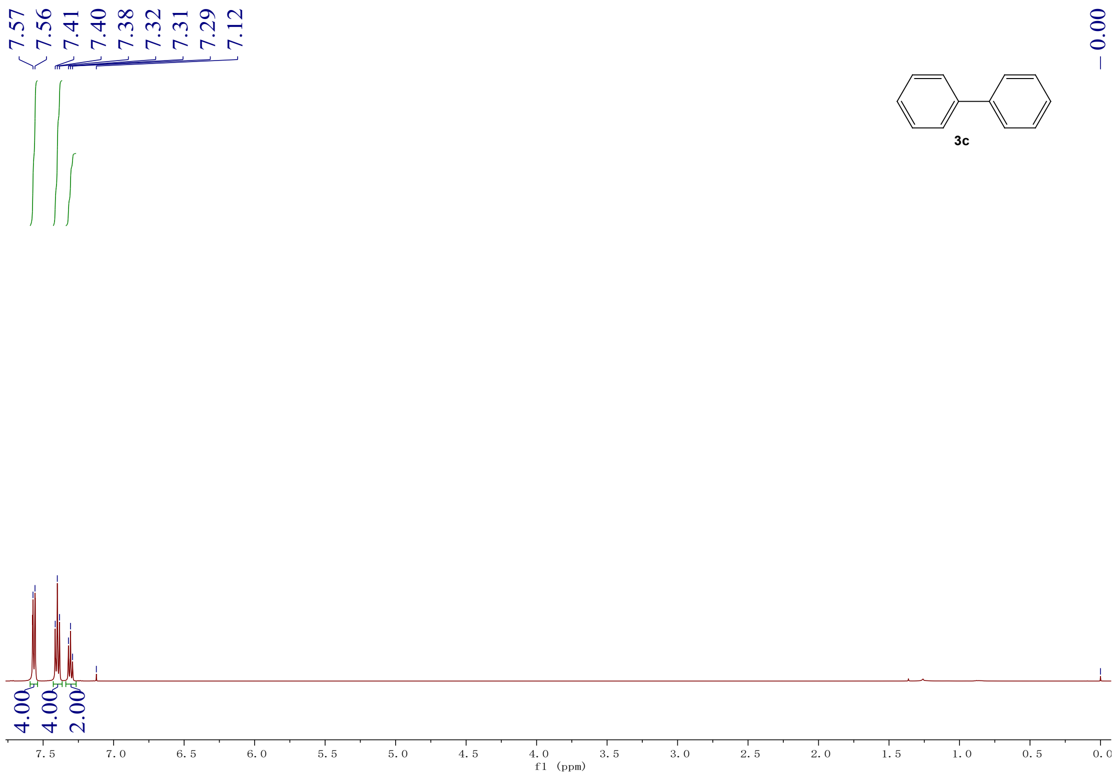
7.57
7.56
7.41
7.40
7.38
7.32
7.31
7.29
7.12
0.00
3c
4.00
4.00
2.00
f1 (ppm)

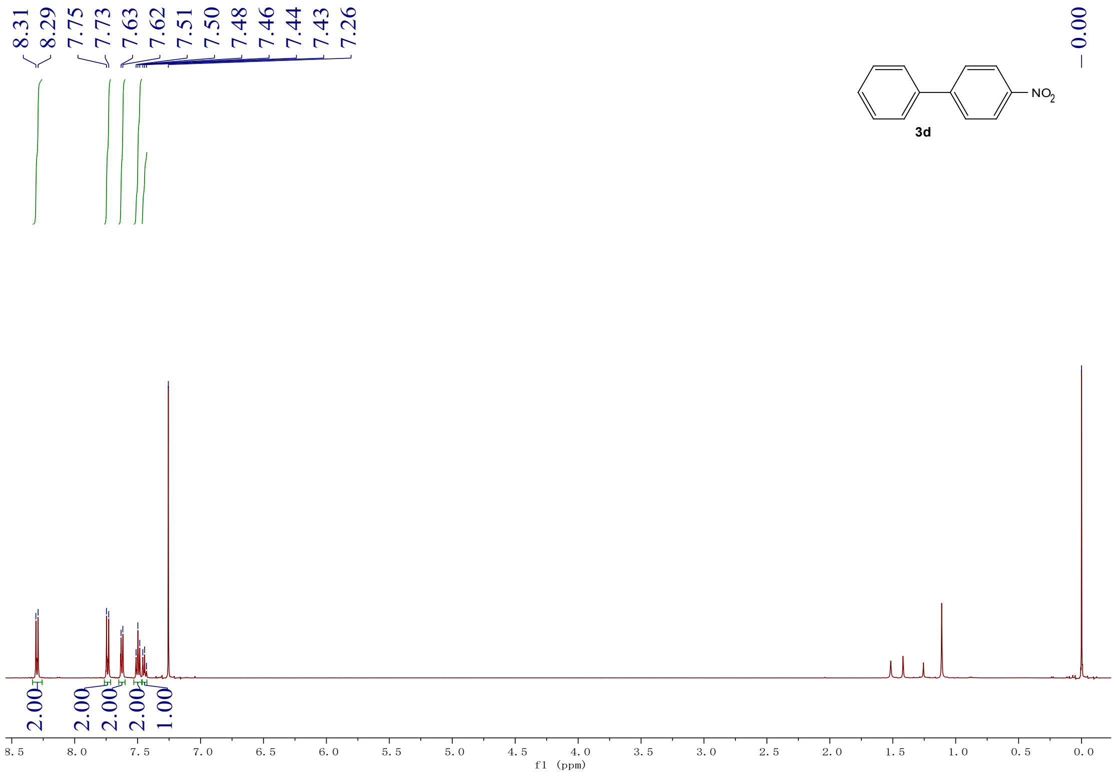
8.31
8.29
7.75
7.73
7.63
7.62
7.51
7.50
7.48
7.46
7.44
7.43
7.26
0.00
NO2
3d
2.00
2.00
2.00
2.00
1.00
f1 (ppm)

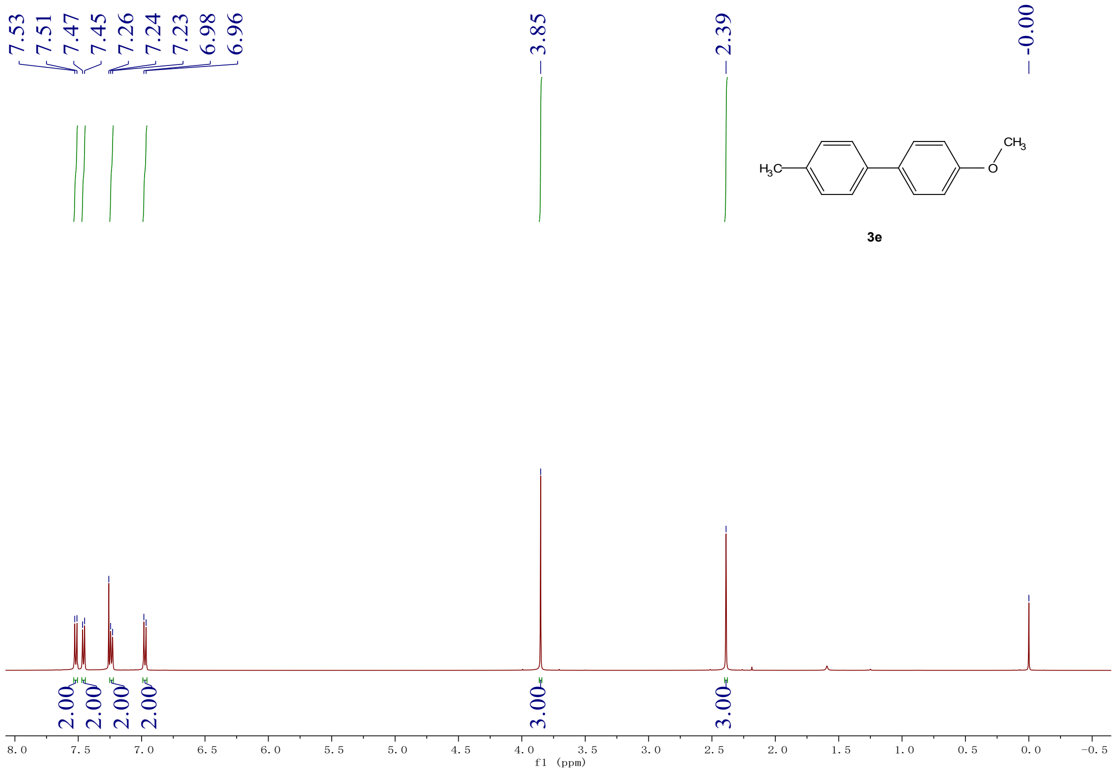
7.53 7.51 7.47 7.45 7.26 7.24 7.23 6.98 6.96
3.85
2.39
-0.00
H3C
CH3
O
3e
2.00 2.00 2.00 2.00
3.00
3.00
8.0 7.5 7.0 6.5 6.0 5.5 5.0 4.5 4.0 3.5 3.0 2.5 2.0 1.5 1.0 0.5 0.0 -0.5
f1 (ppm)

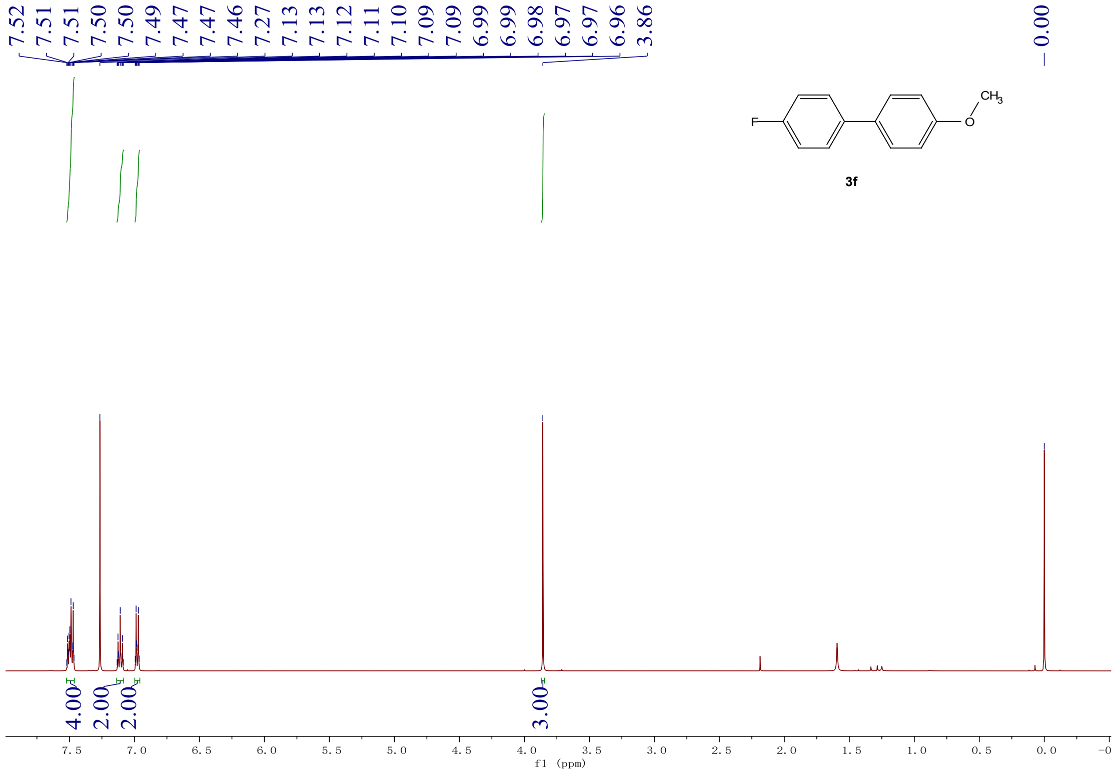
7.52 7.51 7.51 7.50 7.50 7.49 7.47 7.47 7.46 7.27 7.13 7.13 7.12 7.11 7.10 7.09 7.09 6.99 6.99 6.98 6.97 6.97 6.96 3.86
0.00
F
CH3
O
3f
4.00 2.00 2.00
3.00
7.5 7.0 6.5 6.0 5.5 5.0 4.5 4.0 3.5 3.0 2.5 2.0 1.5 1.0 0.5 0.0 -0
f1 (ppm)

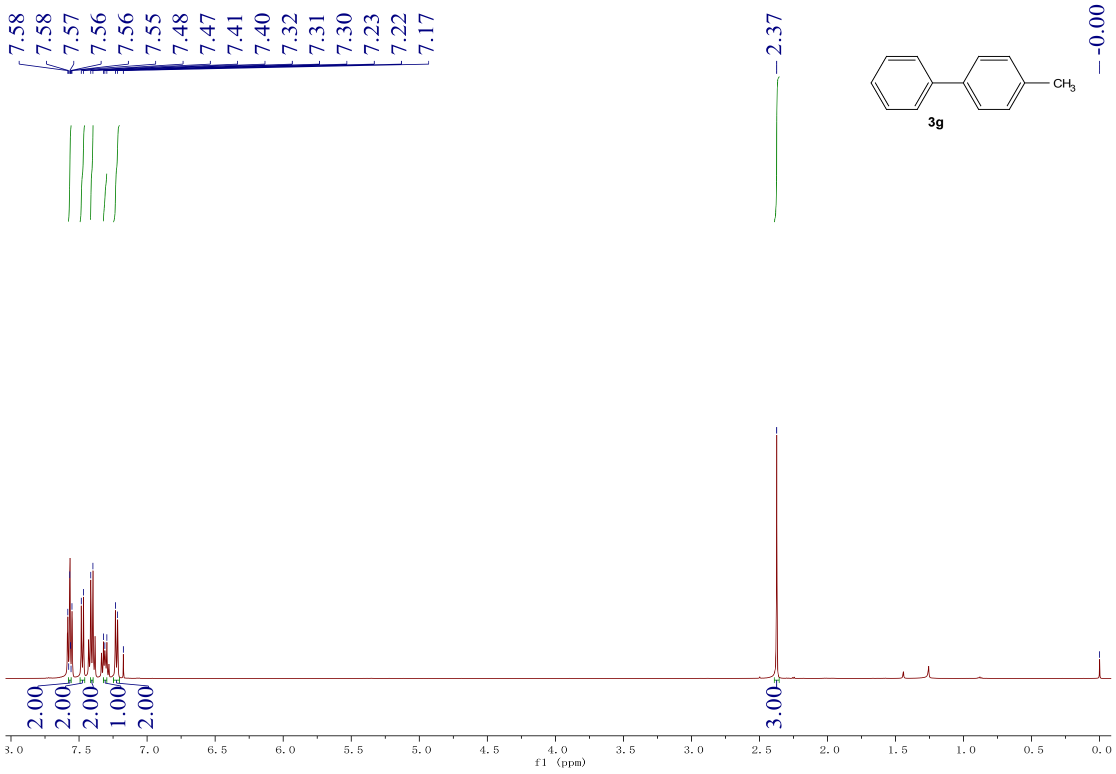
7.58 7.58 7.57 7.56 7.56 7.55 7.48 7.47 7.41 7.40 7.32 7.31 7.30 7.23 7.22 7.17
2.37
-0.00
CH3
3g
2.00 2.00 2.00 1.00 2.00
3.00
f1 (ppm)

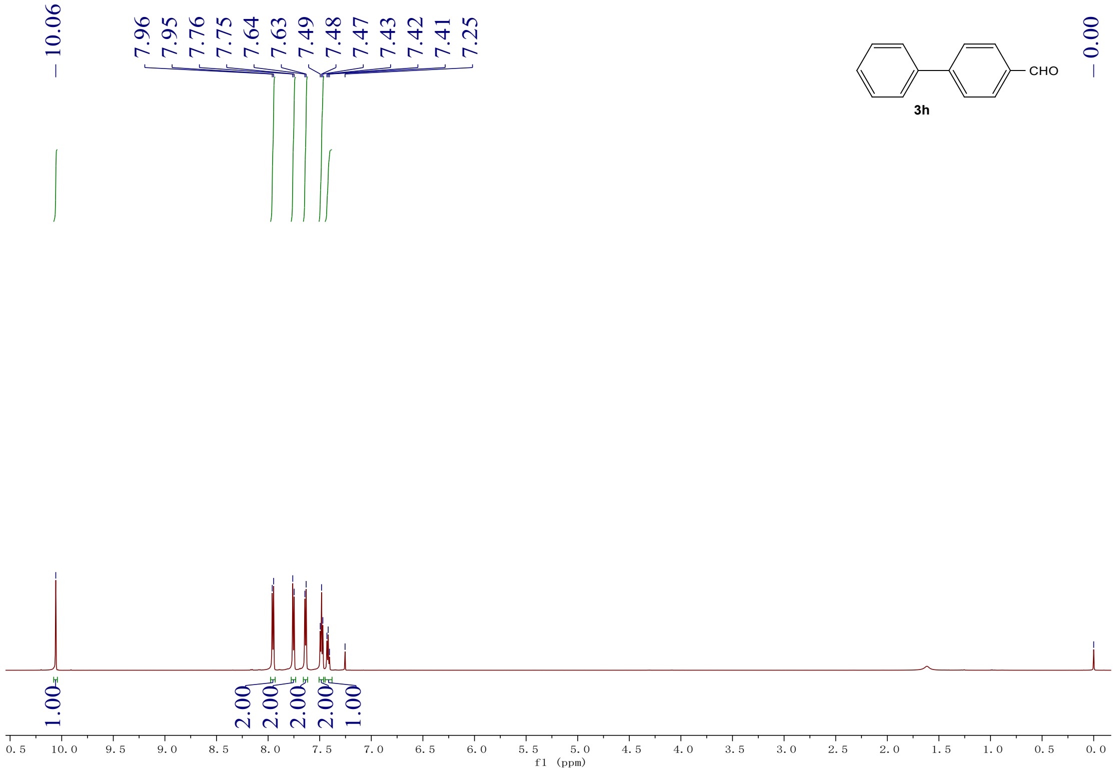
10.06
7.96 7.95 7.76 7.75 7.64 7.63 7.49 7.48 7.47 7.43 7.42 7.41 7.25
0.00
CHO
3h
1.00
2.00 2.00 2.00 2.00 1.00
f1 (ppm)

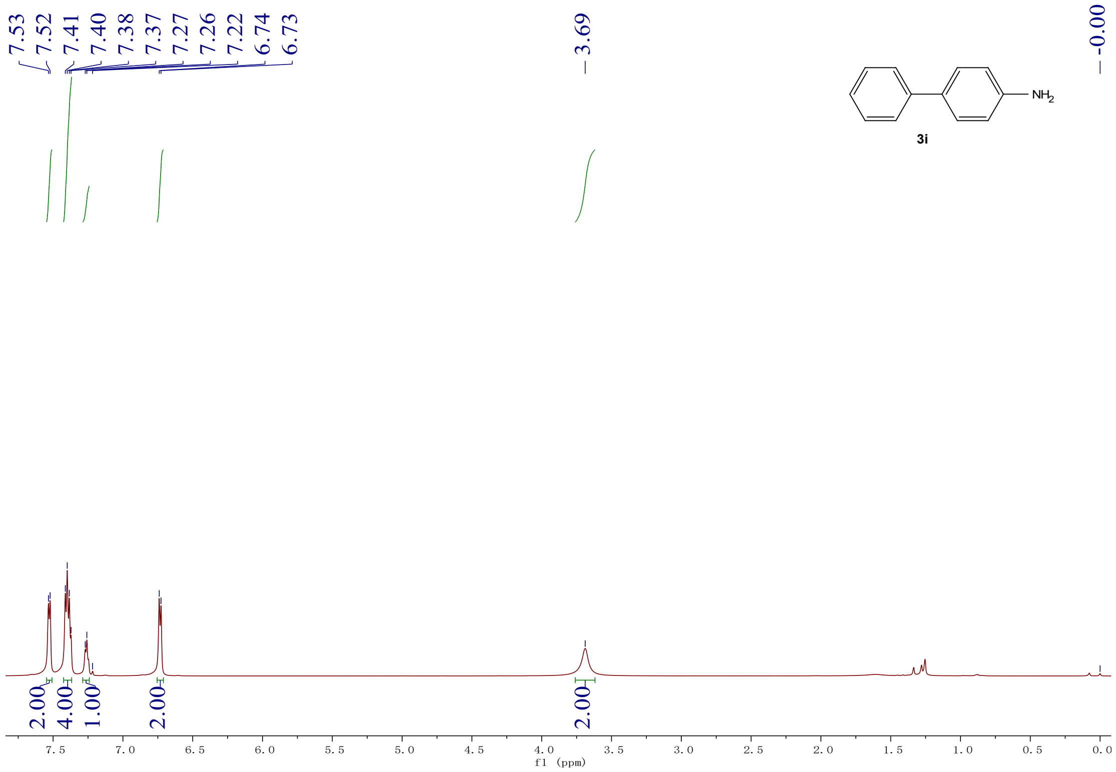
7.53
7.52
7.41
7.40
7.38
7.37
7.27
7.26
7.22
6.74
6.73
3.69
-0.00
NH2
3i
2.00
4.00
1.00
2.00
2.00
7.5 7.0 6.5 6.0 5.5 5.0 4.5 4.0 3.5 3.0 2.5 2.0 1.5 1.0 0.5 0.0
f1 (ppm)

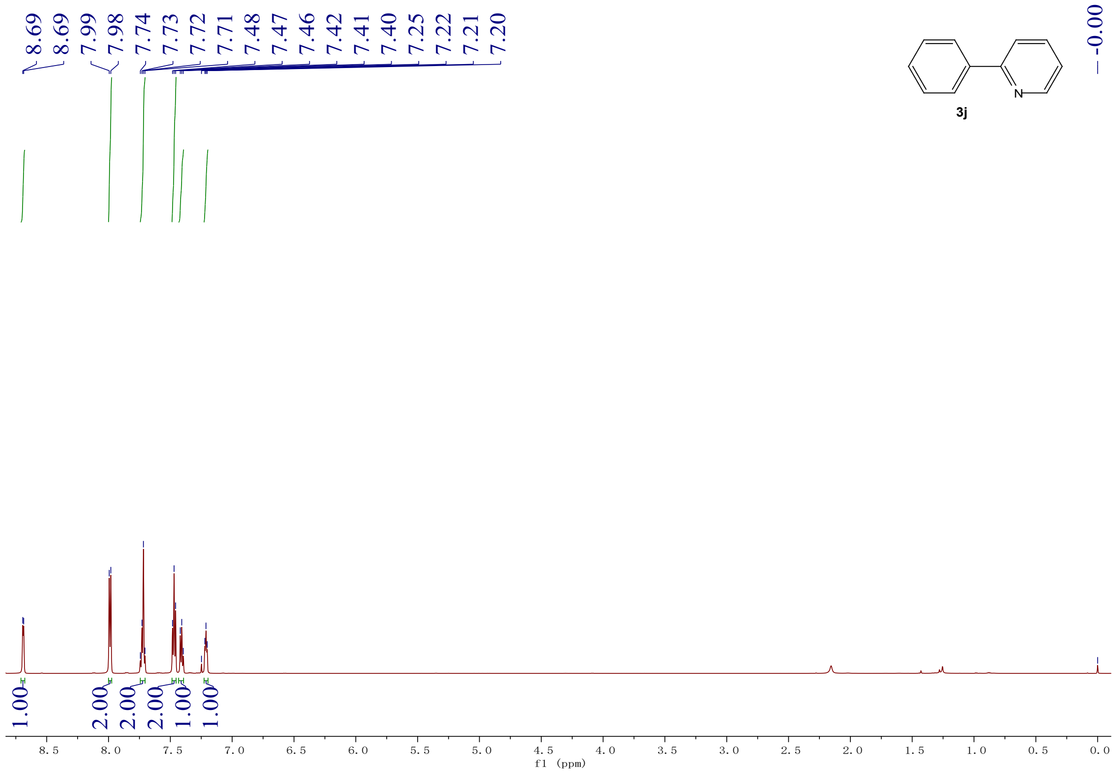
8.69
8.69
7.99
7.98
7.74
7.73
7.72
7.71
7.48
7.47
7.46
7.42
7.41
7.40
7.25
7.22
7.21
7.20
-0.00
N
3j
1.00
2.00
2.00
2.00
1.00
1.00
8.5 8.0 7.5 7.0 6.5 6.0 5.5 5.0 4.5 4.0 3.5 3.0 2.5 2.0 1.5 1.0 0.5 0.0
f1 (ppm)

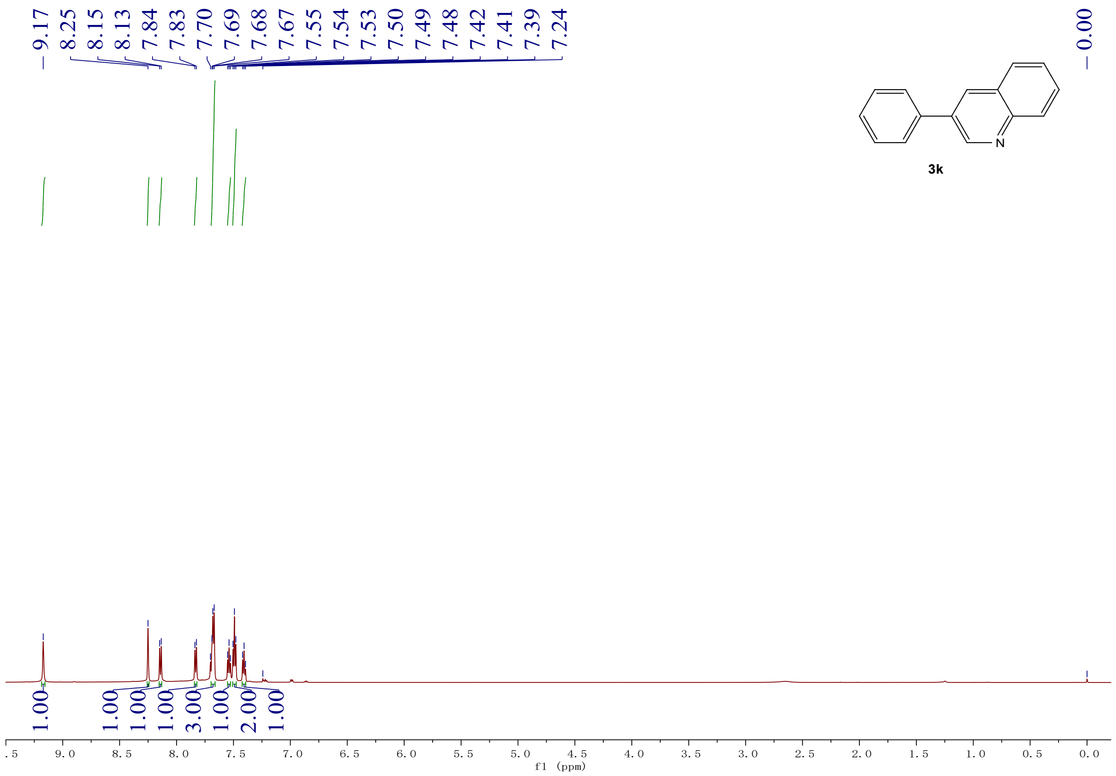
9.17
8.25
8.15
8.13
7.84
7.83
7.70
7.69
7.68
7.67
7.55
7.54
7.53
7.50
7.49
7.48
7.42
7.41
7.39
7.24
0.00
N
3k
1.00
1.00
1.00
1.00
3.00
1.00
2.00
1.00
f1 (ppm)

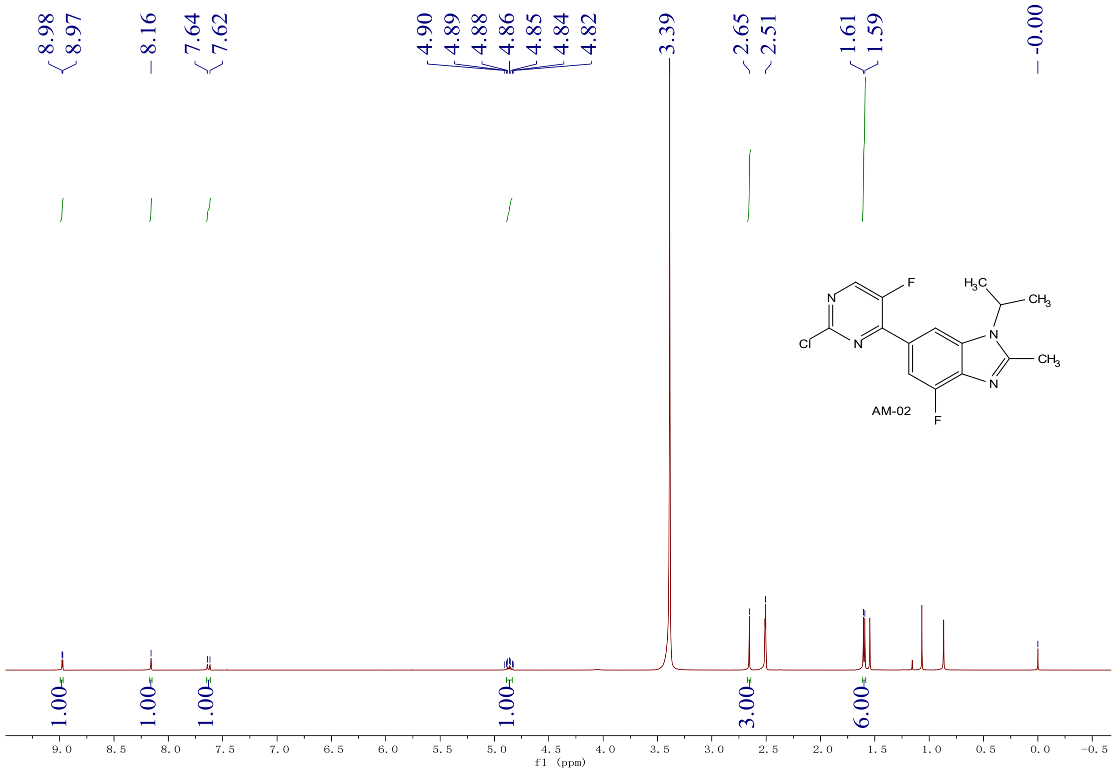
8.98
8.97
8.16
7.64
7.62
4.90
4.89
4.88
4.86
4.85
4.84
4.82
3.39
2.65
2.51
1.61
1.59
-0.00
F
H_3C
CH_3
N
N
N
Cl
N
CH_3
N
F
AM-02
1.00
1.00
1.00
1.00
3.00
6.00
f1 (ppm)
